# Recent advancements in NIR spectroscopy for assessing the quality and safety of horticultural products: A comprehensive review

**DOI:** 10.3389/fnut.2022.973457

**Published:** 2022-10-12

**Authors:** R. Pandiselvam, V. Prithviraj, M. R. Manikantan, Anjineyulu Kothakota, Alexandru Vasile Rusu, Monica Trif, Amin Mousavi Khaneghah

**Affiliations:** ^1^Physiology, Biochemistry and Post-Harvest Technology Division, ICAR –Central Plantation Crops Research Institute, Kasaragod, Kerala, India; ^2^Department of Food Engineering, National Institute of Food Technology Entrepreneurship and Management, Sonipat, Haryana, India; ^3^Agro-Processing and Technology Division, CSIR-National Institute for Interdisciplinary Science and Technology (NIIST), Trivandrum, Kerala, India; ^4^Life Science Institute, University of Agricultural Sciences and Veterinary Medicine Cluj-Napoca, Cluj-Napoca, Romania; ^5^Animal Science and Biotechnology Faculty, University of Agricultural Sciences and Veterinary Medicine Cluj-Napoca, Cluj-Napoca, Romania; ^6^Food Research Department, Centre for Innovative Process Engineering (CENTIV) GmbH, Stuhr, Germany; ^7^Department of Fruit and Vegetable Product Technology, Prof. Waclaw Dabrowski Institute of Agriculture and Food Biotechnology-State Research Institute, Warsaw, Poland

**Keywords:** cultivar authentications, damage detection, maturity, rapid method, spectroscopy, texture

## Abstract

The qualitative and quantitative evaluation of agricultural products has often been carried out using traditional, i.e., destructive, techniques. Due to their inherent disadvantages, non-destructive methods that use near-infrared spectroscopy (NIRS) coupled with chemometrics could be useful for evaluating various agricultural products. Advancements in computational power, machine learning, regression models, artificial neural networks (ANN), and other predictive tools have made their way into NIRS, improving its potential to be a feasible alternative to destructive measurements. Moreover, the incorporation of suitable preprocessing techniques and wavelength selection methods has arguably proven its practical feasibility. This review focuses on the various computation methods used for processing the spectral data collected and discusses the potential applications of NIRS for evaluating the quality and safety of agricultural products. The challenges associated with this technology are also discussed, as well as potential future perspectives. We conclude that NIRS is a potentially useful tool for the rapid assessment of the quality and safety of agricultural products.

## Introduction

Agro-based processing industries are trending toward the production of fresh and minimally processed commodities. In this context, new processes and products are now available on the market, driven by consumer interest. Agricultural products are the main raw material for many food industries (1, 2). They are highly perishable in ambient conditions and thus have a limited shelf life, which can be extended under refrigerated storage conditions. Because fruits and vegetables cannot be stored for a long time due to their susceptibility to deterioration, particularly microbial and chemical spoilage, the various processes in the post-harvest supply chain, such as grading and sorting, should be completed immediately after harvesting.

The use of proper analytical methods to ensure the quality and safety of end products is necessary before processing and throughout the post-harvest supply chain (3). However, conventional methods for detecting defects in fruits and vegetables have several disadvantages: The evaluation of raw fruit quality, cultivar authenticity, and damage to the products pose challenges to The evaluation of the quality of the raw fruit and the authenticity of the cultivar, as well as the assessment of any damage to the product, pose challenges to collecting good quality inputs from farmers or wholesale dealers. Many kinds of damage cannot be detected with a visual inspection alone, thus rendering the process of selecting high-quality products insecure. Conventional testing methods, such as sampling from big lots, are time-consuming and expensive (4–7). Moreover, a significant amount of product is often destroyed during conventional testing and sampling.

Specific techniques are needed to, for example, detect sun scaling in apples and weed out the defectives, as such injuries do not have any chemical treatments (8). Apples can also have internal browning issues that are not visible on the surface (9), thus again ruling out the possibility of visual detection. Similarly, fruits are often kept in refrigerated conditions to extend shelf life, but this poses a risk of causing chilling injury, which cannot be detected by peel color or any other aspect of external appearance (10). Therefore, the standard practice of local markets is to determine the price of fruits and vegetables based on their physical attributes, estimated quantitatively and qualitatively (11).

Non-destructive analytical methods thus play a vital role in overcoming the challenges of conventional laboratory methods. In this aspect, near infrared spectroscopy (NIRS), combined with predictive algorithms, is best suited to assessing product quality and damage detection and for identifying cultivars (12).

**Figure 1:**
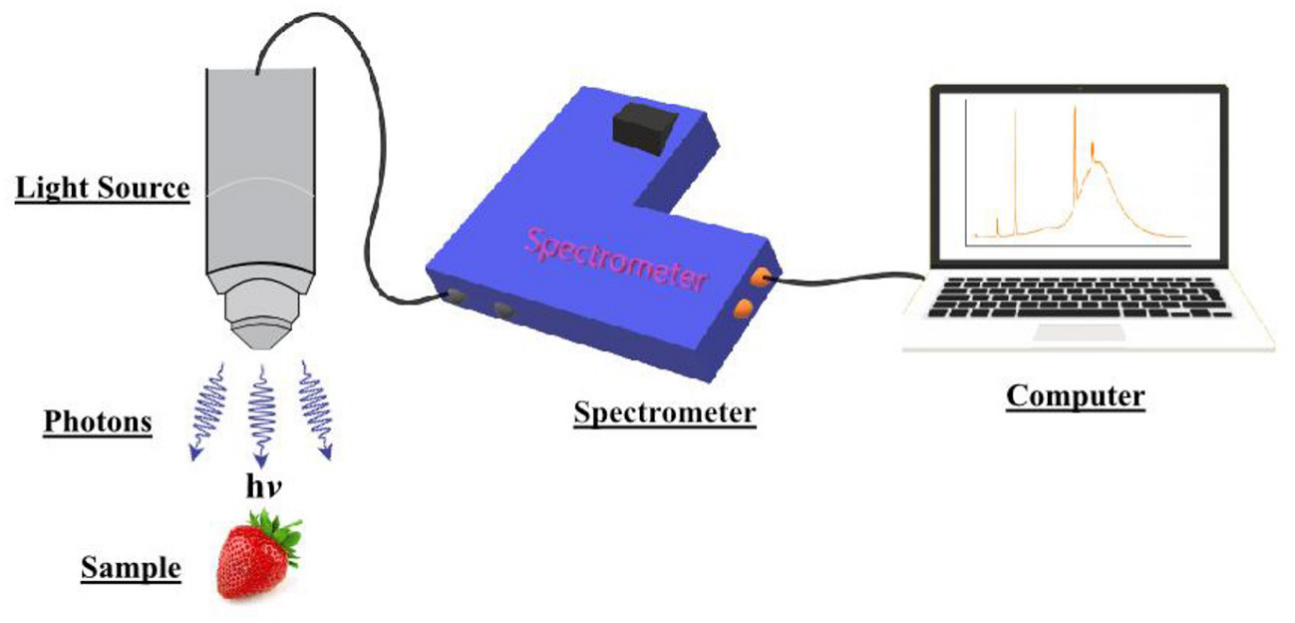
Spectral data collection unit.

**Figure 2:**
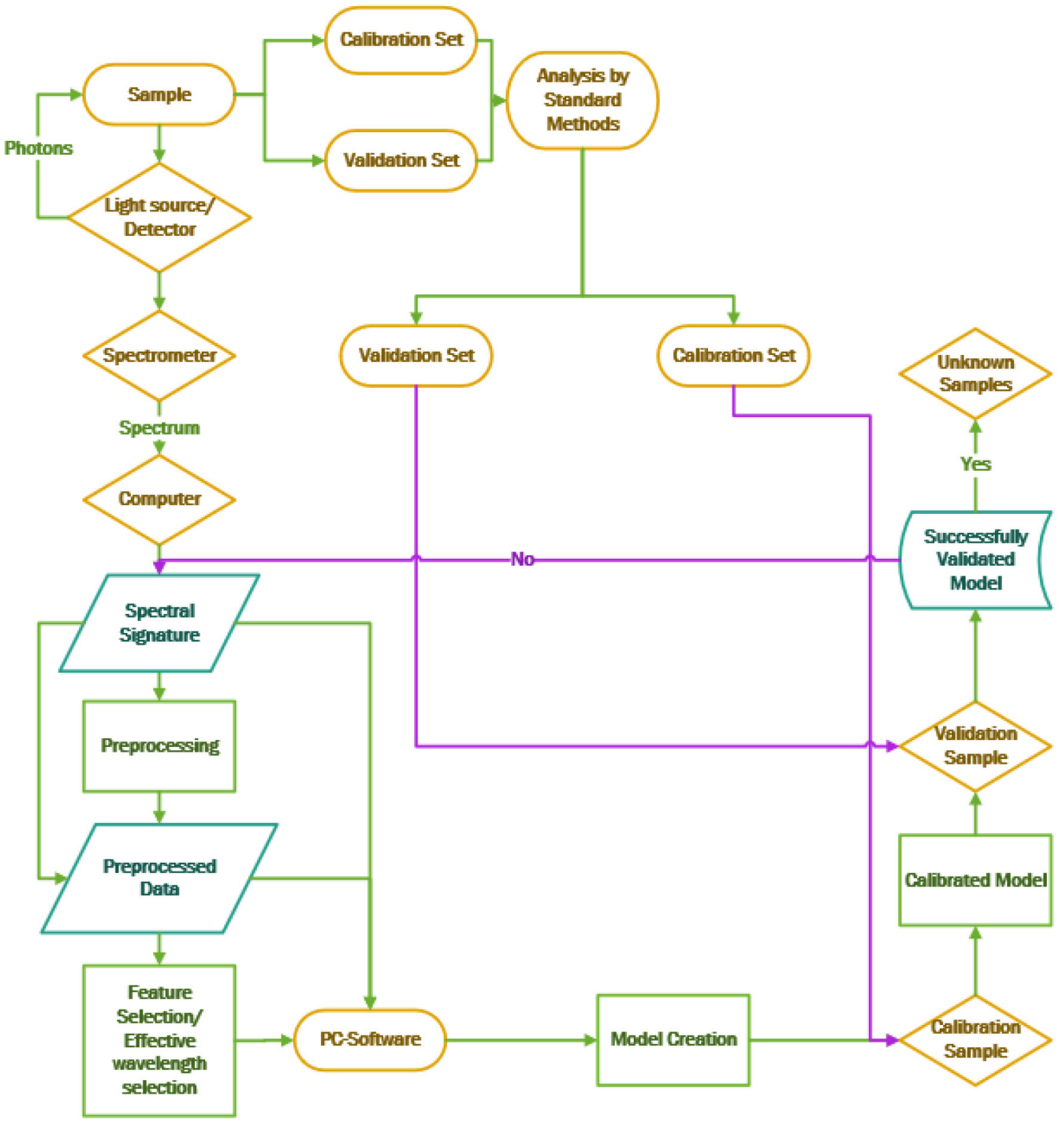
Flow chart depicting the NIRS modeling process.

A NIR spectroscopic system consists mainly of an interconnected light source, spectrometer, and computer, as shown in Figure 1. (This diagram shows the simplest representation possible, and does not account for a more practical NIRS system). The light source emits photons in the infrared region, which come into contact with the sample, and their further interactions create vibrations or stretching (13) in the molecules in the interior of the sample. These vibrations create a spectrum dependent on the properties of the molecule and their corresponding chemical bonds. These spectral profiles are dominant in specific parts of the spectrum. For instance, molecules like chlorophyll vibrate in the 500–750 nm region (8, 9) and O-H bonds in water in the 970–1,150 nm region (9). The spectra have to be analyzed with specific preprocessing techniques to eliminate noise and unwanted and redundant information. Later on, preprocessing techniques is implemented to find the effective wavelength, which spans certain portions of the spectrum in a way that can precisely classify the product, as shown in Figure 2. A properly built model can ably classify the product with little or no mistakes. Care should be taken when designing a NIR spectroscopic system to conduct a perfect analysis, using standard methods, with minimum time delay and high accuracy. The standard error in a laboratory (SEL) may lead to a standard error in prediction (SEP), which should be minimized during quantitative prediction with proper methods.

NIR spectroscopy applications have been reviewed for the processing of cereals processing (14) and seafood (15), for various applications with dairy products (16), and for quality analysis of meat and spices (4, 17). Nevertheless, it is paramount to understand the various applications of NIRS for agricultural products, as these applications could pave the way to preparing high-quality end products from these raw materials. In this context, the present review discusses various NIR spectroscopy measurement techniques and optimization strategies used in agricultural products.

## Applications of spectroscopic techniques

### Quality parameters

Food products, and their reliability, depend on the quality of the products from which they are derived. NIR spectroscopy is one of the best methods for predicting the primary characteristics of these products, *viz*. total soluble solids (TSS), soluble solids content (SSC), titratable acidity (TA), and pH, using spectral signatures and algorithms. The various applications of NIRS for determining the quality of agricultural products are shown in Table 1.

**Table 1:** Uses of NIRS to determine the quality aspects of various agricultural products.

**Agricultural product**	**Spectral range**	**Software package**	**Number of samples**	**Accuracy**	**Findings**	**References**
Apple and apple purees	800–2,500 nm	OPUS v. 5.0, XLSTAT, MATLAB v. 7.5, R v. 3.5.2	240	Classification accuracy of apples = 82% and purees = 88%	Viscosity, cell wall content, dry matter, SSC, and titratable acidity showed best prediction (*R*^2^ > 0.8). Final apple puree conditions were predicted from those of apples.	(18)
Apple	350–2,500 nm	MATLAB 2014a, Unscrambler v. 10.5x	120	SSC: (*R*^2^ = 0.87, RMSEP = 0.55) pH: (*R*^2^ = 0.72, RMSEP = 0.009)	Denoising with wavelet transform before pretreatment was found effective.	(19)
Tangerine	700–1,100 nm	MATLAB v. 7.0	275	94.0% using SSOM	SSOM is a non-linear classifier successfully used to detect MC, SSC, TA, and granulation.	(20)
Elderberry	800–2,500 nm	OPUS v. 7.2, Unscrambler 10.4, Statistica v. 8.0	Fruits from 11 orchards, blended	97.06%	TSS and SSC correlated well with spectral data, and pattern recognition was possible.	(21)
Apple	800–2,500 nm	OPUS v. 7.2	214	TA: (*R*^2^ = 68.17%, RMSEP = 0.12) TSS/TA: (*R*^2^ = 82.62%, RMSEP = 0.43) TSS: (*R*^2^ = 90.93%, RMSEP = 0.61)	Spectral intensities varied according to the spectroscope used. GA significantly improved the prediction quality.	(22)
Hami melons	550–950 nm	Unscrambler v. 9.7	120	RMSEP = 0.95–0.99	SSC determination obtained the best result for the MC-UVE-SPA-MLR combined preprocessing and non-linear prediction algorithm.	(23)
Grapes	800–1,100 nm	Unscrambler v. 10.5	120	TA: (*R*^2^ = 0.716, RMSEP = 0.103) pH: (*R*^2^ = 0.547, RMSEP = 0.395) SSC: (*R*^2^ = 0.971, RMSEP = 0.522)	Considerable variation was observed in connection with various pretreatments. Improvement in calibration results does not always improve validation results.	(24)
Pineapple	740–1,070 nm	MATLAB v. 9.5.0	90	85%	Good prediction accuracy for TSS with RMSEC = 0.95 and RMSEP = 0.84.	(25)
Olives	2,307–2,348 nm	UCal	100 (12 cultivars)	*R*^2^ = 0.964 and 959	Oil content was predicted prior to oil extraction.	(26)
Pomegranate	400–1,100 nm	ParLeS v. 3.1, AvaSoft7	100	TSS: (*R* = 0.95, RMSEC = 0522)	Reflectance mode was better than transmission mode for determining TSS and pH.	(27)
Persimmon	1,000–2,500 nm	NIRware v. 1.2	147	R > 0.75, RPD <1.5	SSC predictions obtained the best result with MSC pretreatment.	(28)
Apple	550–950 nm	Unscrambler v. 10.1, MATLAB 2016a, SpectraSuite, Visual Studio 2010	180	r_p_ = 0.842, RMSEP = 0.453	Keeping the stem-calyx axis vertical, with stem upward, was found to be the best orientation. PLS and LS-SVM were used to create compensation models, CARS and SPA to select effective wavelengths.	(29)
Grape	450–2,500 nm	Vision software, GenStat	120	TSS: (*R*^2^ = 0.896, RMSEP = 0.308) TA: (*R*^2^ = 0.835, RMSEP = 0.066) TSS/TA: (*R*^2^ = 0.812, RMSEP = 0.451)	The balance between sugar and acid was taken as a quantitative parameter, which was correlated with the perception of taste.	(11)
Tomato	400–1,100 nm 900–1,700 nm	MATLAB R2016	600	pH: (r_p_=0.819) SSC: (r_p_=0.800)	Spatially resolved spectroscopy was found effective for agricultural crops with heterogeneous structures and chemical compositions.	(30)
Tomato	930–1,650 nm	SpectraWiz, ParLeS v. 3.1	120	Lycopene: (r_cv_ = 0.840, RMSECV = 2.256) Vitamin C: (r_cv_ = 0.818, RMSECV = 1.087)	MSC combined with first derivative was able to predict lycopene and vitamin C content through the PLS model. The NIR process used here does not afford high accuracy.	(31)
Oranges and grapes	450–2,500 nm	Vision TM v. 3.5.0.0, GenStat	120 Grapefruit 120 Oranges	TSS: (*R*^2^ = 0.927, RMSEP = 0.283) TA: (*R*^2^ = 0.929, RMSEP = 0.017) TSS/TA: (*R*^2^ = 0.958, RMSEP = 0.2) TSS: (*R*^2^ = 0.896, RMSEP = 0.308) TA: (*R*^2^ = 0.835, RMSEP = 0.066) TSS/TA: (*R*^2^ = 0.812, RMSEP = 0.451)	The sweetness and flavor attributes of oranges and grapes were studied. The organoleptic parameter BrimA (Brix minus acids) was evaluated.	(32)

SSC, soluble solid content; RMSEP, root mean square error of prediction; SSOM, supervised self-organizing map; MC, moisture content; TA, titratable acidity; TSS, total soluble solids; GA, genetic algorithm; RPD, residual predictive deviation; MSV, multiplicative scatter correction; MC-UVE, Monti Carlo–uninformative variable elimination; SPA-MLR, successive projection algorithm–multiple linear regression; CARS, competitive adaptive reweighted sampling; RMSECV, root-mean-square error of cross-validation.

A study conducted by Lan et al. on apple quality evaluated the characteristics of the puree produced from the same. The apples' spectral details were used to predict viscosity, cell wall content, dry matter, SSC, and puree product TA. The parameters mentioned above showed *R*^2^ values greater than 0.8, indicating the method's accuracy (18). The study's spectral measurements were carried out in a range of 800–2,500 nm using an automatic sampling wheel with 18 different positions. However, methods using six optical fibers (9) or manual positioning are more realistic and accurate. This may be due to the consistency of the sampling wheel setup. The SSC content determination resulted in an *R*^2^ value of 0.92 due to the homogeneity of the puree product (18).

The algorithms perform best when the product is homogeneous rather than inconsistent. Similar investigations were conducted on calçot onions and on apple purees, and also obtained accurate predictions for soluble solids, glucose, malic acid, and dry matter (33, 34). These studies indicate that the quality evaluation of purees can be successfully performed using spectral analysis.

In these scenarios, the use of partial least squares (PLS) models with TSS prediction resulted in *R*^2^ = 0.95, better than any other parameters (27). However, rheological constants and color values were found to underperform. This may be due to the non-linear nature of rheological variations or the overlapping of spectral bands (33). Prediction of puree characteristics by evaluating the quality of intact apples was challenging and semi-quantitative, suitable for the industrial process (33). NIRS works based on the chemical compositions and photon response; thus, processing commodities and converting raw produce to value-added end product will drastically alter the product's chemical nature. This may be why more latent variables and lower accuracy are obtained in predicting the quality characteristics of processed products.

Khodabakhshian et al. conducted internal quality studies on pomegranates using both transmittance and reflectance modes in the 400–1,100 nm range. Their study evaluated the model performance by using the predicted residual error sum of squares (PRESS) method as a cross-validation technique in the regression analysis. The usual pretreatments, particularly standard normal variate (SNV_ and multiplicative scatter correction (MSC), were carried out, thus accounting for the morphological variation in the pomegranate varieties (27).

Practical case scenarios always need to account for morphological variation. Therefore, while SNV and MSC preprocessing can be used, it may not be the same for all situations. The overall characteristics of both the reflectance and transmittance spectra were similar and formed peaks in the 750 and 970 nm ranges, though more noise was observed in the transmittance spectra (27). The spectra obtained with wavelength depicted irregular spikes all over the data, indicating the noise problems. Distinctive peaks for spectral signature are the preferred method for NIRS analysis. Interestingly, preprocessing techniques remained similar for most of the quality traits irrespective of the crop, demonstrating the potential for efficiency at the point of analysis. Both transmittance and reflectance methods performed similarly, though the latter had a slight upper hand due to its higher penetrating power (21, 35, 36) and more robust system. Thin-peeled fruits such as pomegranate obtained improved results in reflectance mode, suggesting that this method can be used for thick-walled fruits such as coconut, areca nut, and cocoa.

An extensive study on SSC determination conducted by Hu et al. studied various measuring configurations, variable selection algorithms, and classification models with Hami melons. In this context, the calyx model performed the best, which could be due to the higher SSC content in the calyx region (23). This result can be further interpolated to obtain the best prediction results after all preprocessing. The measurement should be taken at the most prominent part of the attribute.

A similar study performed on apples incorporated a greater number of orientations, which resulted in the selection of stem-calyx vertical, with stem upward, as the optimal direction (29). This suggests that whether one orientation performs better than others is due to the prominence of the factor in the oriented region. Various combinations of variable selections and prediction algorithms suggest that the Monte Carlo–uninformative variable elimination–successive projections algorithm (MC-UVE-SPA) attained the best results with all prediction algorithms for SSC determination (R_p_>0.8) (23). Xia et al. found that CARS-SPA-PLS performed best for SSC prediction in apples, obtaining a low root mean square error of prediction (RMSEP) of < 0.573° brix. They also found that the determination of effective wavelength from a global model could help reduce the effect of orientation to a certain limit (29).

Selecting effective wavelengths that correspond well to the regions that classify samples can reduce equipment expenses, as demonstrated by a study on a banana quality evaluation where the lowest possible wavelength window was suggested (37). The effective wavelength of each agricultural product varies and therefore needs to be standardized during spectral analysis. In bananas and apples, this variation of change in the effective wavelength may be due to differences in color and overall composition. We can thus deduce that orientation, effective wavelength, products used, and equipment design should be considered while designing an online measuring system for optimum performance. Various studies have been able to predict SSC content with ${\text{R}}_{\text{p}}^{-}$ > 0.9, obtaining better results than with other factors, such as TA and pH (24). Similarly, the TSS content of pineapple was compared by combining various algorithms (25). This latter study, which used a handheld spectrometer, obtained an accuracy rate of 85% with an RMSEC of 0.95 and an RMSEP of 0.84 (25). This technology offers the best practical results in terms of feasibility and future perspectives, even with using handheld equipment.

Kanchanomai et al. (24) investigated potential rapid evaluation techniques by determining the quality of grapes using SSC, pH, and TA. The SSC prediction obtained an R_p_ value of 0.97, whereas TA (R_p_ = 0.71) and pH (R_p_ = 0.54) were comparatively less accurate. Firmness and seedlessness were also studied, but these factors also had low prediction accuracy. The researchers concluded that a NIR range of 800–1,100 nm could be suitable for predicting internal quality in grapes. Similarly, table grapes were analyzed using various preprocessing techniques and a NIR range of 400–1,000 nm (27, 38). The proper preprocessing technique improved the R_Pred_ from a range of 0.6 to 0.8052 and above when Savitzky-Golay's second derivative (SG2) was applied. This suggests that various factors such as pH, TSS, and firmness can be predicted by applying SG2 preprocessing in table grapes. The better results obtained can be justified using the proper wavelength range and preprocessing technique used on table grapes.

A combination of back propagation neural network (BPNN), generalized regression neural network (GRNN), and particle swarm optimization (PSO) was used in order to determine SSC and total acidic content (TAC) in apples (39). This study focused on developing a hybrid artificial neural network (ANN) model, as mentioned with earlier techniques; this was necessary to overcome the inherent limitations within ANN. The results showed that, during SSC prediction, the hybrid model and BPNN model had nearly the same RMSEC and RMSEP values (<0.6 and <0.7, respectively), whereas the GRNN did not perform well (values of >0.6 and >0.9, respectively). However, during TAC determination, the GRNN and hybrid models had low RMSEC and RMSEP values (<0.1), whereas BPNN did not perform well (>0.2). This confirms that an adaptation of hybrid models indeed increased the versatility of the algorithms to determine various quality attributes rather than having them to be changed with the parameter.

In terms of online determination methods, various systems have been explored for use with apples, mangoes, and bananas. Proper orientation of the product, as indicated in the previous finding, proved to be one of the key challenges facing the online system design. In an effort to address this challenge, the use of the CARS-SPA-PLS model after SGS was studied and was found to be effective in apples (29), with an r_p_ > 0.8. A similar system of MCARS-SPA-PLS was suggested for online apple prediction with higher accuracy. These algorithms can handle biological variability and tackle orientation issues during prediction. Online systems were found highly suitable for squash (40) and juice products, irrespective of the orientation challenges. This is likely due to the inconsistency in the fruits and homogeneity in the value-added products.

Highly perishable produce such as banana needs rapid quality analysis measures. An online conveyor system for the same was designed that took TSS, pH, dry matter (DM), and acid-brix ratio (ABR) into account (37). During the validation phase, corresponding *R*^2^ values of 0.81, 0.78, 0.78, and 0.87 were found, indicating highly accurate prediction results. The study also selectively carried out preprocessing for both pH and ABR, which improved the prediction results. The accuracy of online detection was higher for apples, as the combination of algorithms 'successfully managed the difficulties of product orientation. Therefore, similar combination algorithms need to be adapted for online systems, given the challenges of performing these measurements with precise orientation.

### Textural properties

Texture is one of the key factors influencing the quality, feel, and appearance of agricultural products. The most common parameters used to measure the textural quality of fresh produce are firmness and penetrometer readings (41, 42). Some studies have analyzed a wider variety of parameters, *viz*. fracture force, hardness, apparent modulus of elasticity, compressive energy (43), initial firmness, average firmness, rupture force of peel, rupture distance, penetration energy, and penetration force (42). These parameters can distinguish varieties (41), as different species and biological conditions have unique ranges. It is also worth noting that some studies have opted to use a universal testing machine (UTM) rather than a textural analyzer for evaluating the firmness and penetration depth of more oversized objects.

The summary of the various applications of NIRS for measuring the textural qualities of agricultural products is depicted in Table 2. Sánchez et al. (44) analyzed spinach texture using a handheld NIR device connected to a micro-electrical mechanical system (MEMS). The researchers used a simple handheld device for NIR measurement, thus demonstrating the utility of compact, practical technologies over more complex, high-cost instruments. The response of the NIR spectroscopy to CH, OH, and NH bonds and their absorbance patterns creates crests and troughs (41) in the spectral signature, which is the base of analysis. It was observed that the use of the handheld spectrometer resulted in an *R*^2^ value nearly equal to 1. The commodities used in the experiment were taken from different farms in Spain. Practices like this (i.e., that aggregate a greater amount of varieties and biological variations) allow for better calibration and data processing, which in turn contributes to the building of more robust prediction systems and, ultimately, yields better results.

**Table 2:** Uses of NIRS to determine the textural properties of agricultural products.

**Agricultural product**	**Spectral range**	**Software package**	**Number of samples**	**Accuracy**	**Findings**	**References**
Mango	800–2,500 nm	OPUS v. 7.0.129	85	*R* = 0.7–0.75	Penetration parameters, firmness, and rupture force were predicted.	(42)
Spinach	1,600–2,400 nm	WinISI II v. 1.50	149	$R_{\text{cv}}^{2}$ = 0.74	Consumer acceptance parameters, dry matter, and textural properties were identified.	(44)
Tomato	800–2,500 nm	OPUS	30	*R*^2^ = 0.7–0.97 RMSECV = 0.4% Brix	SSC content was predicted using PLS analysis. The textural prediction was accurate.	(41)
Tomato	950–1,650 nm	SPSS 13.0, PermutMatrix v. 1.9.3, Unscrambler v. 9.7, MATLAB 2017	90	$R_{\text{p}}^{2}$ = 0.85–0.966 RMSEP < 0.5	Raw spectra outperformed preprocessed ones. ELM obtained the best prediction over PLS and SVM.	(45)
Kiwi	800–900 nm	ImageJ, RGS-AvaCam v. 3.7.0, MATLAB R2016b, Unscrambler v. 9.7	116	*R*^2^ = 0.68–0.77	Firmness was influenced by similar gray pixels. PLS give slightly better results than ANN.	(46)
Olive	1,100–2,300 nm	SNAP 2.03, Unscrambler v. 9.7, SigmaPlot v. 10.0	100	*R*^2^ = 0.8–0.99	A large data set can account for several factors and thus achieve accurate prediction. A rapid and inexpensive technique was used.	(47)
Pear	380–1,030 nm	Spectral Image System, Unscrambler v. 9.7, ENVI v. 5.0, MATLAB v. 8.1	135	$R_{\text{p}}^{2}$ = 0.89–0.92	Scanning speed > 1.5 m/s is needed for real-time application. Deep learning using SAE-FNN was performed.	(48)
Pistachio	200–1,100 nm	AvaSoft7, ParLeS v. 3.1	81	*R*^2^ = 0.754–0.91 RMSEP = 0.253–26.049	The SNV model performed better than MSC. Among textural parameters, fracture force was predicted the most accurately.	(43)

SSC, soluble solid content; RMSEP, root mean square error of prediction; RMSECV, root-mean-square error of cross-validation; PLS, partial least squares; ELM, extreme learning machines; SVM, support vector machines; ANN, artificial neural network; SAE, stacked auto encoders; FNN, fully connected neural network; MSC, multiplicative scatter correction.

This assertion is supported by findings from other studies, an investigation of methods for determining tomato texture in which tomatoes in similar maturity groups gave distinct clusters in a scatter plot (41). Distinct clusters will not account for the various maturity stages and varieties present in the sample; therefore, the sample size should be increased to create a better performing model. The textural tests were performed using a universal testing machine rather than a textural analyzer (44, 47). The food matrix tested for puncture force, toughness, stiffness, fracture point (44), and firmness (47). Maximum force to puncture the leaf (r^2^cv = 0.67; RPDcv = 1.72), toughness (r^2^cv = 0.62; RPDcv = 1.62), stiffness (r^2^cv = 0.69, RPDcv = 1.79) and the displacement of the probe necessary to fracture each leaf (r^2^cv = 0.62, RPDcv = 1.61) (44). Similarly, a firmness measurement performed on olives recorded a high *R*^2^ value of 0.997, indicating excellent-quality predictions of this parameter (47). There is a significant improvement in *R*^2^ value with suitable preprocessing, as illustrated by a study on pistachio kernels comparing raw data and various preprocessed data (43).

The selection of the preprocessing method to be used with textural analysis depends on the parameter under study and the instruments used in the measurement. A study conducted by Mohammadi-Moghaddam et al. (43) was able to model better-performing PLS models for textural analysis after various pretreatments. It is worth noting that pretreatment is not always necessary, and that better results were obtained in determining the firmness of pears (*via* PLS regression) without preprocessing (48). Direct comparison between different pretreatments to determine the best choice is not possible, as it depends on several factors.

In olives, firmness is understood to correlate with maturity and oil content (47) due to the relationship between oil and dry matter. As these factors are mechanically and structurally connected, defining the mouth feel *via* NIRS can offer a non-destructive method of measurement. Although firmness prediction is a bit complicated, one study on olives obtained readings of 1,248, 1,449, 1,758, 1,917, 1,990, and 2,238 nm at peak regions using PLS regression, thus delivering high correlation and low error rates (47). A similar study using cherry tomato resulted in a firmness prediction of *R*^2^ = 0.966 (45). The algorithm for the extreme learning machine used in this study works by optimizing the hidden layers in the cross-validation step, thus solving practical problems (45).

Compression and penetrometer tests were performed together on tomatoes by Camps and Gilli (41), with the compression test demonstrating better prediction performance (*R*^2^ = 0.85–0.97). This observation is further supported by a study conducted on juicy stone fruits that found that the compression test was significant as the fruit matured (49). A textural analysis on tomatoes revealed that there are about 7–10 latent variables for compression parameters and anywhere between 3 and 8 for penetrometer parameters (41), and that data clustering may be the reason behind better *R*^2^ results. However, although clustered data can improve *R*^2^ values, the RMSE values may not differ much.

Studies on the correlation between texture and quality in mango suggest that, based on the *R*^2^ value, the parameter should either be used for rough screening or for thorough evaluation. On this basis, one study found that the parameters of pulp penetration force, peel rupture force, and penetration energy in the pulp can be used for rough screening (42). The authors also discussed the relationship between textural parameters and other factors such as maturity, storage time, and processing stage. Processing operations such as roasting can also cause a decrease in moisture content, resulting in more reflectance (43); similarly, a difference in amplitude of peak has been observed in pistachio kernels after processing. Camps and Gilli (41) studied three tomato varieties with 90 samples outperformed this study on the same parameters, although the sample size was 80 on a single variety. These results confirm both the highly variable characteristics of tomatoes and the research gaps regarding these characteristics. Yu et al. (48) proposed a deep learning model with a stacked auto-encoder (SAE) and fully connected neural network (FNN), where SAE was used to extract the features which were given as FNN input. An investigation conducted on pear fruit compared the PLS regression with a deep learning algorithm, and significantly better results were obtained for SAE-FNN (48). Deep learning predicted the *R*^2^ value in the range of 0.9, whereas the PLS regression could only reach values near 0.8 (48). Experiments on firmness prediction in kiwi fruit found that, on average, PLS (*R*^2^ = 0.77) performed better than ANN (*R*^2^ = 0.72) (46).

### Variety/cultivar identification and authentication

Authentication and traceability throughout the supply chain are essential, especially where technologies such as block-chain methods are implemented. Today's consumers are focused on authenticity; they demand traceability and strict quality controls in production. In recent years, NIRS, along with classification algorithms, has been integrated into supply and production chains to meet these demands.

The development of models that can discriminate between different genotypes of apples (50–53), bell peppers, (54), mangoes (55), pears (56), potatoes (57), and mulberries (7) using the NIR spectrum is summarized in Table 3. In practical terms, the sorting of these commodities should be done on a fast-moving conveyor system, but designing a spectrum-collecting system for this environment is troublesome. Because even agricultural products of the same variety can have different surface properties, obtaining multiple spectral samples, as large as 250 readings (7, 55, 59), is necessary. The sample size and the number of measurements needed are determined by the heterogeneity and complexity of the sample, such as whether the product is sliced (59). The heterogeneous nature of the sample is accounted for by techniques such as taking three or four different types of measurements; by the use of 120 and 90-degree rotations (54, 58); and by the use of fabricated fruit holders (59, 61) or by using the arrangement in the spectrometer. Standard NIR models require whole fruit cover scanning using arrangements of diode-array instruments (44), the integration of the sphere around them to recover information that is otherwise lost (52), and methods to keep the distance from fruit to measurement probe constant, irrespective of the size (50).

**Table 3:** Summary of variety and cultivar identification for various agricultural products using NIRS.

**Agricultural product**	**Spectral range**	**Software package**	**Number of samples**	**Accuracy**	**Findings**	**References**
Bell pepper	1,600–2,400 nm	Win ISI II v. 1.5, MATLAB 2015a	394	88.28–91.37%	Preliminary screening using SSC and dry matter was a success. The importance of SEP and SEL was discussed.	(54)
Mulberry	909–1,649 nm	PLS Toolbox v. 6.21, MATLAB R2009	468	84.1%	*Dendrobium officinale Kimura et Migo* (DOK) was distinguished from *Dendrobium devonianum Paxt* (DDP).	(7)
Apple	1,000–2,500 nm	MATLAB 7.11, Antaris II System	180	74.44%	Among PCA, PCA+LDA, SDA, and DPLS, SDA was found to have better performance for feature extraction.	(58)
Apple	1,000–2,500 nm	Fiber Optic Solids cell, NIRWare Unscrambler,	410	77.9%	Classification of apples according to various terrain types.	(51)
Tangerine, red cabbage, cornichons, kale and applesauce	1,100 nm and 2,100 nm	PAS LABS v. 1.2, SIMCA v. 14.1	15	99%	NIRS prediction was possible for commodities kept inside glass. OPLS-DA outperformed PCA and PLS-DA.	(59)
Potato	964.13–1645.01 nm and 2502.50–16666.67 nm	SpectralCube, OPUS v. 7.2, PLS-toolbox v. 8.6, Unscrambler v. 10.1, MATLAB R2017b	240	RP = 0.954 RMSEP = 0.421	A PLSR model was used to find the degree of doneness and predict the variety.	(57)
Apple	300–1,100 nm	ModelBuilder, R Statistical software	640	*R*^2^ values were 0.90 and 0.92 and RMSE were 0.67%.	Individual models for cultivars performed better than the combined model.	(53)
Mango	1,200–2,200 nm	Unscrambler	1,310	Alphonso and Banganapalli (99.07%, 99.58%), Dasheri and Malda (98.37%, 94%)	A distinct score plot allowed for more accurate classification.	(55)
Apple	400–1,021 nm	Ocean View, MATLAB R2014b	300	SPA-SVM 85.83% SPA-ELM 95%	Among BPNN, SVM and ELM models, ELM performed better. Feature selection with SPA combined with ELM produced better results than PCA.	(60)
Pears	350–1,800 nm 350–1,000 nm 1,000–1,800 nm	Unscrambler v. 9.7	110	*R*^2^ 0.90–0.92 RMSEP 0.23–0.30	Feature selection was obtained better with CARS than with MC-UVE and SPA. CARS-MLR and CARS-PLS accurately determined SSC.	(56)
Apple	1,000–2,500 nm	MATLAB R2014a	208	98.1%	Geographical region had a significant effect on SSC. CARS feature selection and PLS-DA had good prediction accuracy.	

SSC, soluble solid content; SEP, standard error in prediction; SEL, standard error in laboratory; PCA, principle component analysis; LDA, linear discriminant analysis; SDA, stacked denoising autoencode; DPLS, dynamic partial least squares; OPLS, orthogonal partial least-squares; PLS-DA, partial least-squares discriminant analysis; PLSR, partial least squares regression; BPNN, back propagation neural network; SVM, support vector machines; ELM, extreme learning machines; SPA, successive projection algorithm; CARS, competitive adaptive reweighted sampling; MC-UVE, Monti Carlo–uninformative variable elimination; SPA-MLR, successive projection algorithm–multiple linear regression.

Most researchers are using the existing spectrometer system directly, and therefore more emphasis has been given to the classification methods. A broader range of photometers and a larger sample size can significantly improve the model (54). Broader models are useful for obtaining unique peaks in the spectrum that can discriminate between varieties. Because fluctuations in temperature and light play a vital role in creating the NIR model, care should be taken to keep the surrounding environment the same for all measurements (53).

For cultivar prediction, the input data are preprocessed using methods such as multiplicative scatter correction (MSC) (51, 55, 60), EMSC (50, 62), standard normal variate (SNV) (7, 51, 56), detrend (51, 55), normalization (58, 59), Savitzky-Golay (7, 50, 51, 61), and Norris gap (55). These are independent reference techniques for eliminating the unwanted effects of irrelevant information in the spectra (52). Without these techniques, the noise would be learned along with the true calibration data, causing over-fitting (53). The application of the various treatments is based on the corrections required by the scenario, and it is up to the researcher to decide which corrections are suitable. Preprocessing is a necessary step in variety detection because the produce being measured is in raw form, and the noise level can therefore be high. However, it should be kept in mind that the use of smoothing techniques, such as those listed above, to process raw data can result in the loss of valuable information.

The investigation of fruit and vegetable spectra using produce housed in glass containers during preprocessing did not yield better results than raw data, at least in a scenario where a reference-dependent orthogonal partial least squares (OPLS) model contained an integrated OSC filter (59). The filter's effectiveness was evaluated by a response permutation set and performance matrices, *viz*. sensitivity, specificity, efficiency, false negative, false positive, and true negative (59).

A direct spectral comparison may not always be possible due to crossovers and overlapping. Li et al. (60) measured 300 samples of apple and observed spectra between 400 and 1,021 nm, and all of which were largely similar to each other. The observed spectra consisted of various attributes, so dimension reduction techniques such as PCA and the score plot were later used for classification. Dimension reduction was necessary to eliminate irrelevant and redundant spectral variables (56). In the context of cultivar identification, PCA was able to retain 98% of the data for apple cultivation (51), which makes it an ideal feature extraction method.

Jha et al. (55) investigated the classification of apple varieties and obtained 99.07% and 99.58% accuracy for Alphonso and Banganapalli mangoes, respectively. Score plot variance typically decreases from PC1, PC2, and PC3; thus, most researchers use the first two (51) or the first three (50). The variety inherent within the identification process needs a score plot where similar results are grouped or clustered in one area. However, choosing PC2 and PC3 regardless of PC1 can result in better models with a better grouping (54). Variable selection should be made using the genetic algorithm (GA), the successive projection algorithm (SPA), Monte Carlo–uninformative variable elimination (MC-UVE), and competitive adaptive reweighted sampling (CARS), and these selected variables should be combined with PLS or multiple linear regression (MLR) in a later stage for predicting (7, 54, 61). Comparative evaluations of these techniques have suggested that CARS has the best performance, resulting in Rp values near 0.9 and RMSEP values near 0.5 (7, 52, 56). Pattern recognition can also be achieved using machine learning methods such as backpropagation neural networks (BPNN), support vector machines (SVM), and extreme learning machines (ELM). These methods are suitable for identifying the variety and geographical origin of fruits based on the spectrum. The number of factors and groups plays a substantial role in the performance of the model. Reducing the number of possible predictions increases the model's accuracy. In one study of apples from different orchard levels, for example, the models performed well only for classification between valley and mountain—i.e., a 2-variable model (51).

Individual models are more accurate than multivariate models (53), but multivariate models need to be developed in practical scenarios. In cases where individual models are deliberately selected for cultivar predictions, data overfitting and insufficient external validations are possible pitfalls (53). Model development requires a suitable training and testing set, and it is ideal to have the same number of training and testing sets for each of the varieties that need discrimination. If the number of elements is not equal between the training and testing sets, there can be a class imbalance, which can lead to biases within the model. Imbalances are usually dealt with by using under- and oversampling techniques, though other methods are possible; Zimmer and Schneider (59), for example, evaluated a well-handled model without treating class imbalance. Models are trained with data from destructive methods and laboratory evaluation. Therefore, the model's standard error in prediction (SEP) depends highly on the standard error in the laboratory (SEL). Sánchez et al. (54) suggested that SEP values greater than or equal to 5 times the SEL value indicate a model of unacceptable quality. For this reason, in a quantitative prediction, it is better to estimate the repeatability and reproducibility of measurements for better understanding. Most of the research comes with the significant caveat that external evaluation is lacking (53), but evaluation of this kind is necessary for a model used to predict cultivars and varieties from various places.

In the case of variety and cultivar identification, the models are used to classify qualitative data, and this automatically enables a quantitative classification (54), i.e., different cultivation locations confer a set of internal qualities that are unique to each variety (60). Eisenstecken et al. (51) and Li et al. (61) conducted experiments to determine varieties grown at different elevations, where quantitative factors (viz., carbohydrate, fructose, sucrose, glucose, sorbitol, and citric acid) displayed a predictable variation according to the elevation at which the fruit was cultivated. This knowledge, combined with a multi-origin regression model, can predict quality parameters such as pH, firmness, acidity, and moisture (52). Nevertheless, the models created during one season are not suitable for the next season due to natural variation in the environment, which in turn creates variation in crop attributes (53).

### Maturity detection

Maturity detection is one of the main uses of NIR in the context of agricultural products. Due to variations in biological factors, many crops do not have standard maturity indices (63). While there is no way to directly predict the maturity of the fruit, factors such as color, firmness, TSS, TA, and dry matter are often correlated with maturity. Among these parameters, color and the pigment component responsible for color are frequently used for maturity detection. This may be because the maturity of fruit was traditionally determined by analyzing the color with the naked eye. One NIR study of lipophilic antioxidants and firmness in tomatoes found a particular trend in the spectral lines as they approached maturity (64). Although this study is not directly related to maturity prediction, the results suggest the potential of NIR for maturity detection.

Fuzzy logic algorithms are among the most recent technologies introduced for maturity detection. Chen et al. (65) evaluated pomelo maturity using a least square support vector regression (LSSVR) model and PCA algorithm with “fuzzy-optimized” NIR data. Compared to ordinary PCA, the fuzzy transform PCA inhibits original data noise and emphasizes PC (65). The LSSVR model is represented by Equation 1.


(1) $$\begin{array}{l} Y=\omega .\emptyset \left(x\right)+b \end{array}$$

where ω = matrix of regression weight; φ(x) = kernel function, which is taken as radial basis function (RBF); b = threshold.

The study divided the samples into three categories, viz. calibration, validation, and test. The test samples are not related to calibration or validation; therefore, they can be used to check whether the model is representative (65). The maturity parameter, which reflects color (L, a, b) values, is thus obtained using the model.

The various applications of NIRS for maturity detection are presented in Table 4. A study of maturity detection in grapes using PLS modeling included variable factors such as cultivar, location, ripeness level, and season (66). Compared with a study that considered only varieties collected from a single vendor for classification, validation, and testing, the former approach is more practical and industry-relevant. Creating the model on the 2016 variety and validating it with the 2017 variety (66) proved the model's long-term robustness. Obtaining the best possible prediction results for each of the parameters under study required the use of different preprocessing techniques; some examples are MSW+MSC for TSS and SNV for TA (66).

**Table 4:** Uses of NIRS for the maturity detection of various agricultural products.

**Agriculture produces**	**Spectral range**	**Software package**	**Number of samples**	**Accuracy**	**Findings**	**References**
Grape	800–2,500 nm	OPUS v. 7.2	267 + 71	R${}_{\text{p}}^{2}$ = 0.28–0.77, RMSEP = 0.14–7.83	TSS and TA parameters can discriminate between grape varieties. Consumer needs and sensory parameters are correlated.	(66)
Avocado	940–1,798 nm	Latentix v. 2.12	> 10,000	*R*^2^ = 0.732, RMSEP = 1.83	Evaluated the performance of a portable NIR device. Conducted external validation with different seasons.	(67)
Mango	306–1,140 nm	Unscrambler v. 10.3	149	R${}_{\text{cv}}^{2}$ = 0.84–0.87, RMSE_cv_ = 139%	PLS prediction worked well for SSC and DM. Firmness had poor calibration.	(63)
Watermelon	802–805 nm	OOIBase32, Unscrambler v. 9.7, MATLAB v. 7.10 R2010b	200	76.7–85.1%	RPP and NDIP techniques were used. Over-maturity was identified.	(68)
Tomato	285–1,200 nm	Model Builder v. 1.1.0.105, SAS v. 9.2		*R*^2^ = 0.67–0.86, RMSCV = 0.64–1.082	Readings of lipophilic antioxidants were only partially correct, an destructive measurement techniques were used. Online measurement is possible with suitable improvements.	(64)
Banana	400–1,000 nm		45	*R*^2^ = 0.89, RMSE = 0.000598	Chlorophyll content was predicted. Skin color was related to maturity.	(69)
Pomelo	400–2,500 nm		168	R_T_ = 0.893–0.912, RMSE_T_ = 0.87–7.28	Fuzzy logic application in LSSVR with RBF kernel. Online quality determination with Vis-NIR is possible.	(65)
Pomelo	380–2,520 nm	QualitySpec Pro		R_p_ = 0.913–0.997, RMSE_p_ = 0.59–5.82	MOPLEC preprocessing and MWPLS were combined to form a robust model. Color parameters L*, a*, and b* were close to LabMeas values.	(70)
Blueberry	400–2,500 nm	Sisvar 3.0, IBM SPSS Statistics 19	300	R = 0.714–0.970	I_AD_ variations and anthocyanin levels were related.	(71)
Apple	200–1,100 nm	Spectrawiz	170	93.27–99.62%	The color and maturity of apples were correlated. Prediction was carried out using ANN-SA and was successful.	(72)
Kiwi	729–975 nm	SAS v. 8.2, F-750 Model Builder	100	*R*^2^ = 0.73, *R* = 0.48–0.74	Dry matter and SSC can be estimated from NIRS. The edible quality of baby kiwi fruit was predicted from the unripe stage.	(73)
Mango	700–990 nm	Spectrasuite, XLSTAT 2014.1, SAS v. 9.4	1,200	70–72%	Maturity can be determined using NIR measurements of DM and TSS.	(74)
Pineapple	740–1,070 nm	MATLAB v. 9.5.0	90	100%	Organic and conventional cultivation was perfectly determined.	(25)

TSS, Total soluble solids; TA, Titratable acidity; RMSEP, Root mean square error of prediction; RMSECV, Root mean square error of cross validation; PLS, Partial least squares; SSC, Soluble solids content; DM, Dry matter; RPP, Ratio of intensity between peak1 and peak2; NDIP, Normalized difference intensity of peak; LSSVR, Least square support vector regression; RBF, Radial basis function; MOPLEC, Modified optical path length estimation and correction; MWPLS, Moving window partial least squares; ANN-SA, Artificial neural network simulated annealing algorithm.

The maturity of kiwi fruit and mango was predicted by measuring dry matter (DM) content and solid soluble content (SSC) (63, 73). Although SSC can be used for maturity detection by measuring the starch in the mesocarp converted to sugar, this method is not recommended in mangoes (63). An investigation of avocado fruits predicted their maturity by estimating moisture content (MC), but this method did not fit well due to the poor spectra obtained (67). The experiment underperformed at first due to poor peel penetration, but improved once the peel was removed. This suggests that NIR cannot be used for all purposes. Evaluations carried out with and without flesh had the same number of latent variables, but the former's *R*^2^ value was 0.6, which later improved to 0.8 (67). In light of these issues, the corresponding challenges in fruits with similar properties to avocado need to be addressed, and proper methods need to be formulated.

**Figure 3:**
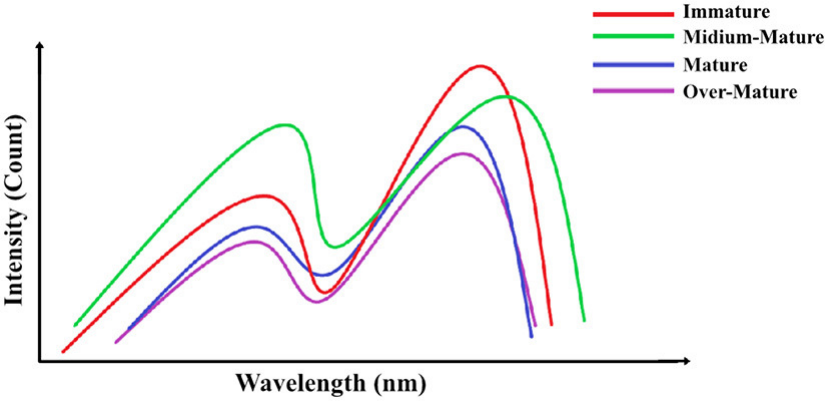
The spectral patterns of products at various stages of maturity.

Temperature has a direct impact on spectral data due to the changing behavior of chemical compounds. Therefore, temperature should be stabilized before measurement, as has been done with kiwi fruit spectra (73). Kim et al. (73) also evaluated the use of Ca-Chitosan coating on kiwi fruit and its effect on maturity. The application of NIRS was able to increase SSC content due to a decrease in the ripening rate (73). The model was able to predict maturity, with *R*^2^ values of 0.73 (73), an acceptable result. Fascinatingly, these results show that applying coatings can change the spectral behavior due to the interaction between photons and the new chemical compounds. However, due to the use of algorithms and processing techniques, all coated fruits with the same compounds will show spectral differences only in other variables, such as SSC and DM, given that a uniform application is performed (Figure 3).

Investigations of banana maturity by Saputro et al. (69) correlate chlorophyll content with maturity determination. A combination of PCA, PCR, and SVM achieved an RMSE value of 0.000598%, implying a good performance. The chlorophyll bands are situated in the 680–700 nm range, which was estimated with the help of NIRS (69). In mangoes, SSC determination was in the range of 699–999 nm and DM at 699–981 nm, which resulted in *R*^2^ = 0.874–0.87 in cross-validation (63).

Ribera-Fonseca et al. (71) evaluated the potential of a non-invasive tool to predict the fruit harvest date using maturity parameters. The device measures an index of absorbance difference (IAD), which has a high correlation with fruit quality parameters (71). This approach could not make use of any conventional algorithms; therefore, it relies on IAD values only. Maturation studies of mangoes by Polinar et al. (74) created a classification model that predicts mature and immature fruit. It was based on days after flower induction (DAFI) by setting the threshold as floaters <25%. Prediction of the ripe stage using these techniques was able to detect artificially ripened fruit with a bland taste (74). These innovations can be used to create auto sorting, identify higher-quality fruits, and prevent fraud in markets.

Watermelon maturity prediction was carried out using the ratio of peak (RP) method. The RP is used when two peaks have opposite intensity variation as the fruit matures. In watermelon, after calculating the ratio of the intensity of peak 1 and peak 2, the normalized difference intensity of peak (NDIP) was used to determine the maturity stage of the fruit (68). The SPA successive projection algorithm (SPA) was used for feature extraction, and least square support vector machines (LS-SVM) was used for classification purposes (68). The plot of the ratio of intensity between peak 1 and peak 2 (RPP) vs. maturity stages was able to distribute the fruit according to different maturity stages (68). Thus, the RPP alone distinguished various maturity stages, without performing PCA or LDA to plot with the highest variations. The correct classification rate (CCR) was highest for corrected RPP values (88.1%) with NDIP (85.1%) and lowest for LS-SVM (76.7%) (68). A high level of CCR is desirable, but at the same time, error parameters also need to be evaluated, which was not mentioned in this study.

Many studies rely on SSC and DM models that use PLS to predict maturity. Taking the spectral analysis to the next level requires the use of different applications, such as those that are used in robotics or that can process video. One study of Fuji apples applied NIR spectral analysis to video frames to detect maturation (72). Rather than the use of quantitative data and correlation, expert panelists were chosen to determine maturity. The processing computer used in the study had “entry-level” specifications by 2020 standards, thus demonstrating the affordability of the analysis, in contrast to “high-end” specifications. The classification model used was ANN combined with simulated annealing (SA). The SA sends out different vectors to ANN and finds the most suitable ANN structure (72). The method performed well, with accuracy rates above 90%, using the wavelength regions of 535–560 nm, 835–855 nm, or 950–975 nm (72).

Chen et al. (70) determined Shatian pomelo maturity by correlating color and maturity levels. Rather than conventional preprocessing methods, the multiplicative effect correction (MOPLEC), which corrects the change in optical path length, was used (70). This technique performed better in comparison to Savitzky-Golay smoothing and increased the signal-to-noise ratio (70). The preprocessing was followed by a moving window partial least square (MWPLS), which found a high signal wavelength combination for PLS modeling (70). MWPLS varies the location and number of adjacent wavelengths to find the optimum combination for PLS modeling (70). The study compared the raw data, SG- processed, and MOPLEC-processed data and found that Rv was highest for MOPLEC-processed data, showing on-par or better values than SG-processed data (70). The new techniques were able to perform better overall in predicting the maturity of pomelo from color. Therefore, these techniques need to be explored for other fruits to determine whether color prediction can be improved for them as well.

### Damage detection

Fresh and minimally processed food commodities are in high demand these days, which raises the stakes on delivering commodities with minimal damage. Well sorted and less-damaged products increase consumer satisfaction, too. There is, however, a great deal of variation among horticultural products: Highly perishable commodities such as tomatoes can show the effects of bruises or damage within 48 h (75), whereas apples can take more than 50 days (8) to show any signs of damage on the outer surface. Harvested commodities should be promptly evaluated for damage to receive the commodity and then convert to any value-added products. Due to the short evaluation period, many food researchers and industries use non-destructive techniques such as NIR spectroscopy that can detect damage without any effects on the products (Table 5). Defects such as internal bruising in blueberries can be detected as soon as 30 min after mechanical impact, with an *R*^2^ of 0.7 (76). Because we can estimate the spoilage period and extent of damage, characterizing spectral signatures so as to detect damage or abnormalities could improve crop utilization (75).

**Table 5:** Summary of NIRS applications for damage detection in agricultural products.

**Agricultural product**	**Spectral range**	**Software package**	**Number of samples**	**Accuracy**	**Findings**	**References**
Peach	400–2,500 nm 325–1,100 nm 930–2,548 nm	ENVI v. 4.6, Isuzu Optics	200	Bruise detection = 96.5, Sound sample detection = 97.5	SW-NIR was found more suitable than LW-NIR. Improved watershed segment algorithm used.	(61)
Blueberry	950–1,650 nm	LabVIEW, MATLAB 2016b, ENVI v. 4.7	320	70.8%−100%	Prediction accuracy increased with time. Calyx side bruise is difficult to detect.	(76)
Apple	400–1,100 nm	MATLAB, Neural Network Toolbox v. 4	550	Correlation of prediction = 0.87, SEP = 5.8N	Back-propagation neural network, combined with input ratios of scattering profile, predicted fruit firmness and SSC.	(77)
Olive	1,100–2,300 nm	R v. 3.1.3 SNAP! V. 2.04	744	96%	QDA was found better than LDA.	(78)
Apple	550–1,650 nm	MATLAB R2017a, PLS Toolbox v. 8.6	430	92.9%	The influence of different positioning on spectra acquisition was studied. The equatorial orientation was found best.	(9)
Kiwi	400–1,100 nm	MATLAB R2018a, PLS toolbox v. 8.7	129		Water-soaked tissues have lower potential for damage detection after the SNV process.	(10)
Coconut	900–2,500 nm	OPUS v. 6.5, Unscrambler v. 9.8	202	94.03%	The NIR model performed better than the acoustic method for detecting cracks in coconut shells.	(13)
Apples	350–1,100 nm	Unscrambler v. 10.5.1, MATLAB R2017b, PLS Toolbox	393	*R*^2^ = 0.59	Sun scaling causes changes in chlorophyll content.	(8)
Cherry	350–2,500 nm	RS3, MATLAB 2011a, ENVI v. 4.6, ViewSpec Pro v. 6.2.0, Origin8 SR0, Unscrambler X10.1	300	93.3%	Color, firmness, and SSC were consistent with the Vis-NIR reflectance. LS-SVM combined with SPA detected bruises within the sample.	(79)
Peach	300–1,150 nm	OMNIC v. 8.2, R Studio, SPSS v. 22.0, OriginPro 2017	840	R_p_ = 0.71–0.92, RMSEP = 0.17–20.34	Good correlation between physiological indicators and absorption spectra. GA-PLS performed the best.	(80)
Tomato	5,555.56–11,111.11 nm	MATLAB 2016		83–97%	PCA-LDA identified *G. candidum* infection.	(75)

SW-NIR, Shortwave near infrared; LW-NIR, Longwave near infrared; SSC, Soluble solid content; QDA, Quadratic discriminant analysis; LDA, Linear discriminant analysis; SNV, Standard normal variate; LS-SVM, Least square support vector machine; GA-PLS, Genetic algorithm partial least square; PCA-LDA, Principal component analysis and linear discriminant analysis.

Damage predictions are conducted by spectral examination of the outer skin, known as the epidermis. Because the epidermis is the “interface” of the fruit structure, any unfavorable conditions can cause small changes (75) that may not be apparent in the 500–900 nm range (i.e., visible to the naked eye) (79). These changes can, however, be detected using NIR spectroscopy. However, some changes, such as oxidative browning, can be detected by the human eye due to the significant time lag involved (76).

In terms of collecting input for spectral processing, various configurations have been studied, viz. taking measurements in 90-degree rotations (10) or 120-degree rotations (13), contacting the epidermis (8), using 360-degree measurements (9), and taking multiple measurements at different points and averaging the values (75). In a comparison of apples held at different orientations for measurement, orienting apples along the equatorial region showed the highest potential for defect detection (9). This may be because this orientation exposes a greater amount of curved surface area, which allows for more sensitive measurements. However, the equatorial alignment yielded the worst results in detecting chilling injury in kiwifruit; in this context, results were better with alignment at the stylar end, likely because chilling symptoms begin at the end of the fruit (10). It was observed during a hyperspectral analysis of blueberries that the calyx end was misclassified as bruised; these data needed to be excluded to reduce the false positive rate and reduce the RMSEP of 0.1 to 0.13 (76). Therefore, the design of NIR systems to measure fruit characteristics should take into account both the defect to be detected and the algorithm used for analysis.

A 6-fiber individual measurement system performed better than the conventional single-point measurement system (9), as accuracy increased from 0.8% to 1.7% when average spectra were used. There is no single method best suited for measurement, because all measurements are post-processed using different methods. Re-orienting can take up a large amount of time needed for developing a commercial online system, which is not suitable. During the training, care should be taken to avoid any outliers, which can affect the classification accuracy by causing overfitting problems. Outliers, in the context of damage detection, refers to commodities that are severely damaged. These cause the PCA and LDA algorithms to underperform. An analysis of sunscald apples followed this procedure to avoid the highly damaged ones, thus increasing the classification accuracy (8). In practical terms, this is possible because severe defects are visible to the naked eye.

An experiment conducted to detect cracked coconut shells found that a PCA plot itself can be used to find the outliers in cases where it is impossible to observe the shell inside the husk (13). The PCA analysis will yield more relevant results once it is run only with effective wavelengths and with outliers eliminated. A PCA analysis of bruising on peaches, with effective wavelengths of 781, 816, 840, 945, and 1,000 nm, yielded a PC4 with high-contrast bruised area (60). When selecting the optimal wavelength, the successive projection algorithm (SPA) follows an iterative process, adding one more wavelength with each step, which reduces the complexity of dealing with the whole spectrum (79). During a bruise analysis of cherry, SPA with 3 variables had an accuracy rate of 96.6%, while the full spectra with 2 variables had a 96% accuracy rate with less computational load (79). A lower computational load, i.e., a shorter analysis time, is essential for the design of an inline system. During the bruising analysis, it was found that even slight damage to the control samples can cause a partiality in the classification model (76). One more caveat is the need to use an independent set of crops from a different region or season. The influences of spatial and temporal variability are still not accounted for by the usual classification algorithms, due to high biological variability. As seen in the detection of sunscald in apples from different years, the RMSEP for prediction was three times that of the calibration (8). The variability caused by biological variation, temperature fluctuation, and measurement positions can be accounted for, to an extent, by using classification models such as least square support vector machines (LS-SVM) (76).

The classification of defect detection is usually done subjectively, i.e., not quantitatively, meaning that human error can occur (10). When the spectra are used for chemometric or qualitative measurement, they should be immediately assessed after the spectral measurement to reduce errors caused by the increase of time (80).

The detection regions are explored by comparing the sound and damaged fruits using their spectral reflectance curves. These depict the reflectance vs. wavelength plot for both samples. Due to the changes caused by damage, there will be significant variation in some parts of the plot between sound and damaged produce, and these differing bands are used for classification. As far as the classification is considered, sound fruits are more homogeneous, and therefore easier to classify, than damaged ones (61). A consistent variation in the spectral sign is desirable for accurate separation (52). This approach also reduces redundant information and the time needed for processing. For SW-NIR and visible NIR, wavebands are selected instead of wave points, to reduce the complexity (80).

In an analysis of bruising on peaches, it was found that SW-NIR (7,801–1,000 nm) was more efficient than LW-NIR (1,000–2,500 nm) due to better contrast difference in the bruised area (60). During the analysis of HIS, wave points and regions of interest (ROI) are selected for analysis (61, 76), and they also provide the spatial distribution of the bruises. Upon correlating the significant effect of the epidermis and spectral curve on research, we observe that pigments such as carotenoids and chlorophyll bands provide promising regions of variation, *viz*. at around 670 nm for the chlorophyll band (8, 10, 52), 550 nm for the anthocyanin band (79), and 740 nm for the carotenoid band (8). Defects in fruits with lower chlorophyll content may not show significant chlorophyll band variation, as is the case with internal browning in apples (60). Evaluating specific chemical bonds and determining the wave points is significantly easier than characterizing TSS or SSC, each of which contains several compounds (80) and is thus tedious to identify on a spectral reflectance plot. The wavelength range of SSC for cherries was 900–2,500 nm, a large waveband that needs further analysis (79).

One study of bruising in blueberries followed the analysis of the cut piece's pixels to evaluate the degree of bruising (76), which was more accurate than a subjective human evaluation. Supplementary techniques such as inverse adding doubling (IAD) can also be conducted on the optical properties of water-soaked tissues (10). The water absorption peaks were found to be at the 970 nm and 1,200 nm regions. A bruise capable of causing cell membrane rupture will undoubtedly differ significantly in this region (76).

The traditional methods followed the PCA and PLS methods, which project all variables to reduce dimensions and increase separability, respectively. During PCA analysis, the first few PC are selected for classification due to their feature-rich plots. Nevertheless, an analysis of bruising on peaches found that PC4 had the best bruise distinction, with retention of effective wavelengths (61). Therefore, while performing a PCA, the resultant plot should be observed to check whether it fits the purpose. To further enhance the efficiency, non-linear optimizations should be carried out, such as the genetic algorithm (GA), where the feature selection follows the natural selection procedure (80). One study of an online system suggested that the band ratio method can be used as a real-time and cost-effective method, with accuracy rates above 90% (76).

### Detection of microbial/fungus contamination

Horticultural crops are prone to microbial contamination. Here, we explore early detection of microbial and fungal contamination using NIRS. Early non-destructive detection within 24 h, in the case of *Botrytiscinerea* and *Colletotrichum acutatum*, can stop cross-contamination and economic loss (81). Studies conducted by Matulaprungsan et al. (82) on cabbage contamination by *E. coli* and *S. typhimurium* created artificial conditions and inoculated samples of shredded cabbage for various time periods to explore the potential of NIR analysis (Table 6). During spectral analysis, this method showed a spectral shift due to the leaking from cabbage cells and the growth of bacteria (82). This shifting behavior was treated using the SG-derivative method (82), followed by tomato pathogen analysis (83). Spectral analysis revealed that the shredded samples had better separation between inoculation times than the ground samples (82), which makes the former the method of choice for analysis. Shredded cabbage, therefore, resulted in a higher r-value (*r* = 0.91–0.95), indicating reliable prediction (82). *E. coli* detection in lettuce also resulted in an accuracy rate of 100% in the validation set using NIR spectroscopy and partial least-squares discriminant analysis (PLS-DA) (36). Various concentrations of *E. coli* were able to be classified using five different techniques, *viz*. PLS-DA, SVM, PCA, hierarchical cluster analysis (HCA), and soft independent modeling by class analogy (SIMCA). PCA and HCA trended toward a coarser classification, whereas SIMCA and SVM produced finer classifications into subgroups. The *E. coli* analysis was carried out in baby spinach using the PLS method (35). For contamination studies, outliers need to be eliminated using feature selection techniques such as PCA. In studies carried out to detect *E. coli* and *Z. rouxii*, PCA used to remove outliers (6, 35). In the case of *E. coli* detection in spinach, Q-residual-Hotelling's T2 plot was used to find out the outliers (35). *E. coli* was detected using PLS-DA supervised learning in the 450–994 nm range, with a 100% accuracy rate in the prediction set (35). The model could not predict unsafe samples in the early stages. Therefore, when the cell concentration reached 6.67 log CFU/mL, it was detected as unsafe during statistical analysis (35).

**Table 6:** Summary of NIRS applications for the detection of target microbial/fungus contamination in agricultural products.

**Agricultural product**	**Spectral range**	**Software package**	**Number of samples**	**Accuracy**	**Findings**	**References**
Tomato	550–1,100 nm	Unscrambler v. 10.3, SpectraSuite	45	74–90%	*F. oxysporum* f. sp. *lycopersici, R. solani, B. acillusatrophaeus*, and *P. aeruginosa* infections were detected.	(83)
Strawberry	400–1,000 nm	Unscrambler X v. 10.1, SpectralDAQ v. 2.1 for STATISTICA 10	2,700	97%	*B. cinerea* and *Collatotrichum acutatum* infections were detected. The BNN model exhibited the highest predictive accuracy.	(81)
Kiwi	833–2,500 nm	OPUS v. 5.5, MATLAB 2012a, Libsvm v. 3.20	352	*R*^2^ = 0.961–0.999	*Z. rouxi, Hanseniaspora uvarum*, and *C. tropicalis* infections were detected. The SVM model was on par with the plate counting method.	(6)
Lettuce	350–1,100 nm	SpectraWiz, Unscrambler X10.3	200	87.1–89.39%	*E. coli* ATCC infection was detected. SIMCA and SVM outperformed HCA, PCA. *E. coli* content varied with the chemical compositions, creating non-linear relationships.	(36)
Cabbage	700–1,100 nm	CA Maker, Unscrambler	20 g	R = 0.47–0.91, SECV = 0.45–1.17	*E. coli, S. typhimurium* infections were detected. Shredded leaves were more suitable for detection. Not a directly non-destructive approach.	(82)

BPNN, Backpropagation neural network; SVM, Support vector machine; SIMCA, Soft independent modeling by class analogy; HCA, Hierarchical cluster analysis; PCA, Principal component analysis.

A study on tomato pathogen analysis achieved a classification accuracy rate of 99.3% on the first and second day using PCA followed by an SVM classifier (83). Although higher classification rates were achieved as the mixing of samples began, the SVM classification could not handle multiple factors affecting the same. The high classification rates were able to detect *F. oxysporum* and *R. solani* fungi, as well as bacteria such as *B. atrophaeus* and *P. aeruginosa* (83).

The analysis of liquid samples created problems due to transparency (82). NIR essentially works through photons and their response to corresponding compounds, so as a solution becomes clear, it becomes non-homogeneous and therefore changes the spectral response. In this context, it is best to use the SW-NIR technique for analysis due to its higher penetration power and lesser interference from water bands (83). However, the study on microbial contamination in lettuce suggested that the water band corresponding to 970 nm O-H stretching needs to be accounted for (36). The differences in microbial cell count can cause molecular vibration patterns (36). In studies of solid lettuce samples, preprocessing techniques such as standard normal variate (SNV) and multiplicative scatter correction (MSC) were used to remove scatter effects (36). Elimination of end bands and spectral resolution enhancement can be performed in order to bring greater focus to prominent wavelengths (36). Preprocessing is carried out to avoid noise and the inclusion of unwanted information that does not add to prediction accuracy. A ratio of prediction of deviation between 1.5 and 2.0 indicates well-calibrated models (82).

Fungal infection caused by *B. cinerea and C. acutatum* in strawberry was analyzed using spectroscopy. The analysis extracted regions of interest (ROI) in the spectral range of 450–2,500 nm with 3500 pixels (81). The data were analyzed using four different classification methods, *viz*. backpropagation neural network (BPNN), random forest (RF), naïve Bayes (NB), and support vector machine (SVM) (81). The classification accuracy decreases once the fungal activity is reduced, which was observed after 4 days in *C. acutatum*–infected samples. However, this technology has its challenges, *viz*. problems with reproducibility, recovery, and the negative effects of humidity and temperature (81). The insight into the influencing factors was a new finding, and the results of this study can thus be used to improve techniques through control of the same.

Yeast species tend to cause deterioration of high-sugar products, particularly fruit products. The yeast species *Zygosaccharomyces rouxii*, which displays this behavior, was analyzed by Niu et al. (6) using NIR to perform non-destructive detection. During preprocessing, it was found that either SG smoothing or direct orthogonal signal correction (DOC) can be used (6). Terminologies such as the limit of detection (LOD) and limit of quantification (LOQ) were introduced in this study, and they varied along with various preprocessing and classification algorithms (6). These terms can be treated as the threshold values for performing detection using the model. The model was able to detect *Z. rouxii* according to the existing standards, with an accuracy rate >85% for both the calibration and prediction models (6).

### Challenges and future prospects

NIR is one of the most researched non-destructive methods in the food processing industry due to its fast-paced analysis, minimal need for sample preparation, cost-effectiveness, and non-destructive nature (84). However, like all technological advancements, NIRS also comes with some disadvantages to be tackled, *viz*. standard error in the laboratory (SEL) causing standard error in prediction (SEP); variations in orientation (29); and performance issues due to biological variation (8). Apart from these challenges, which are related to spectral measurements, regression tools, machine learning, and ANN can also cause problems such as overfitting (53), low correlation values (making the method a primary sorter only), pattern recognition problems (39), and a lack of proper knowledge for choosing the best techniques among the plethora available.

Because these problems have been around since the beginning, some solutions have also been found for them. These include testing and optimizing orientation for parameters, like the testing done with SSC determination that determined that keeping the stem-calyx axis vertical, with stem upward, was best due to the presence of SSC in the stem region (29). During model preparation, the conditions need to be perfect to avoid SEL and SEP, because a single error can cause the model to underperform.

Biological variation remains a challenge, especially when testing with another sample of the same variety from the next season. This is primarily because machine learning works on pattern recognition, and every single year, a large number of biological variables change, which alters the parameters of the product. The way to solve this problem is to have a large training set from various seasons, which is, however, practically impossible.

In order to solve this issue, the techniques of the preprocessing and prediction phases need to be modified so as to adapt the pattern; otherwise, every year, the model must be taught from scratch. This is not possible, since perfect conditions would be needed to recreate a model with minimum errors. Neural network–related issues are being tackled using various techniques such as BPNN, GRNN, and PSO, which performed better when it was left individually (39). Although these methods seem to solve the problem to a certain extent, they can increase the computational load. Cloud computing–based models from various parts of the world may become the next solution, since most of the advanced systems are built around such a setup.

However, machine learning aided by ANN seems to be the foremost approach for NIR analysis, since it deals with biological commodities. The extensive variations in agricultural products can be accounted for using neural networks and pattern recognition algorithms. Predetermined algebraic equations and models created on exact mathematics can be expected to fail in this scenario due to their inability to account for complex phenomena underlying the products' life cycles. Whereas, NIRS got its start as a series of offline systems, today's researchers can create online rapid analysis prototypes, demonstrating the current pace of technology.

The research matrix shows that a significant portion of the studies to date have been conducted on apples, and a huge list of agricultural crops are waiting to be explored. Compact handheld systems have been shown to perform satisfactorily with NIRS, spurring hopes for the next era, when these technologies will hopefully be more widely available and more affordable. Many contemporary smartphones come with IR blasters, light detection and ranging (LIDAR) hardware, and many other sensors. Soon, we all might have NIRS scanners with cloud-based models that can also account for biological variations.

## Conclusion

Spectroscopic techniques can create unique signatures for chemicals due to the particular behaviors of various molecular levels. This allows the quantitative and qualitative analysis of agricultural crops and the selection of those products with the most desirable characteristics. Spectrometer data are used to perform regression analysis and machine learning, by which the evaluation of agriculture crops is conducted. What began as offline test measurement is now capable of performing rapid online measurements without destroying the samples. Advances in computation have greatly improved the technology without much economic expenditure. Current methods involve the creation of hybrid models, which can address biological variations to a certain extent by taking the best parts of various techniques and combining them. Apart from this advancement, challenges remain on the technical side, i.e., the need to address the variation of products, data accusation, and model optimization. There is still an urgent need for a compact, non-destructive technology that can characterize a wide variety of horticultural commodities.

## Author contributions

RP: conceptualization, methodology, data curation, formal analysis, and writing—review and editing. VP: visualization, writing original draft, and writing—review and editing. AK: visualization, methodology, writing original draft, investigation, writing—review and editing, supervision, and funding acquisition. MM: visualization, methodology, writing original draft, investigation, writing—review and editing, and funding acquisition. AR and MT: resources, writing—review and editing, and funding acquisition. AKh: conceptualization, methodology, validation, resources, and writing—review and editing. All authors contributed to the article and approved the submitted version.

## Funding

This work is based upon the work from COST Action 18101 SOURDOMICS – Sourdough biotechnology network toward novel, healthier and sustainable food and bioprocesses (https://sourdomics.com/; https://www.cost.eu/actions/CA18101/), where the authors (AR, MT, and AKh) are members of the Working Group (1). SOURDOMICS supported by COST (European Cooperation in Science and Technology). COST is a funding agency for research and innovation networks. COST Actions help connect research initiatives across Europe and enable scientists to grow their ideas by sharing them with their peers – thus boosting their research, career and innovation. We would also like to acknowledge the funding received by AR from the Romanian National Authority for Scientific Research and Innovation, CNCS-UEFISCDI, project number PN-III-P2-2.1-PED-2019-1723 and PFE 14, within PNCDI III. RP was highly grateful to ICAR-AICRP on PHET for financial assistance.

## Conflict of interest

Author MT was employed by Centiv. The remaining authors declare that the research was conducted in the absence of any commercial or financial relationships that could be construed as a potential conflict of interest.

## Publisher's note

All claims expressed in this article are solely those of the authors and do not necessarily represent those of their affiliated organizations, or those of the publisher, the editors and the reviewers. Any product that may be evaluated in this article, or claim that may be made by its manufacturer, is not guaranteed or endorsed by the publisher.
